# Emerging advantages and drawbacks of telephone surveying in public health research in Ireland and the U.K

**DOI:** 10.1186/1471-2458-6-208

**Published:** 2006-08-15

**Authors:** M Boland, MR Sweeney, E Scallan, M Harrington, A Staines

**Affiliations:** 1 UCD School of Public Health and Population Science, University College Dublin, Dublin 2, Ireland; 2 Department of Public Health, Health Services Executive, Dublin Mid-Leinster Region, Ireland

## Abstract

**Background:**

Telephone surveys have been used widely in public health research internationally and are being increasingly used in Ireland and the U.K.

**Methods:**

This study compared three telephone surveys conducted on the island of Ireland from 2000 to 2004, examining study methodology, outcome measures and the per unit cost of each completed survey. We critically examined these population-based surveys which all explored health related attitudes and behaviours.

**Results:**

Over the period from 2000 to 2005 the percentage of calls which succeeded in contacting an eligible member of the public fell, from 52.9% to 31.8%. There was a drop in response rates to the surveys (once contact was established) from 58.6% to 17.7%. Costs per completed interview rose from €4.48 to €15.65.

Respondents were prepared to spend 10–15 minutes being surveyed, but longer surveys yielded poorer completion rates. Respondents were willing to discuss issues of a sensitive nature. Interviews after 9 pm were less successful, with complaints about the lateness of the call. Randomisation from electronic residential telephone directory databases excluded all ex-directory numbers and thus was not as representative of the general population as number generation by the hundred-bank method. However the directory database was more efficient in excluding business and fax numbers.

**Conclusion:**

Researchers should take cognisance of under-representativeness of land-line telephone surveys, of the increasing difficulties in contacting the public and of mounting personnel costs. We conclude that telephone surveying now requires additional strategies such as a multimode approach, or incentivisation, to be a useful, cost-effective means of acquiring data on public health matters in Ireland and the U.K.

## Background

Telephone surveys have been used widely in public health research internationally [1-3]. In Ireland and the U.K., the use of this survey methodology has been dominated by market research companies and has only recently been used for public health based research [4-6]. The use of computer-assisted telephone interviewing (CATI) is becoming more commonplace. This method offers advantages over the telephone-to-paper interview by reducing time-consuming data transfer and potential subsequent error [1,2].

Telephone surveying has a number of advantages over face-to-face interviewing, allowing a geographically dispersed sample, including those living in remote rural areas, to be reached easily. There is concern that fixed line penetration may be lower in more remote rural areas, and in areas of low population density. This requires careful consideration for each country, and for each region, where it is proposed to carry out telephone surveys. A recently published survey of European telecommunications service indicators shows very wide variation in the penetration of fixed line services within European regions [7].

Interview travel time and associated costs are eliminated and interviewers do not have to physically visit study areas. Consequently, concerns about interviewer safety are reduced. Respondents gain perceived anonymity, so telephone surveys may be very useful in collecting data of a sensitive nature [8,9].

Limitations of telephone surveys include lack of representativeness, as households with no landline telephone are not represented. Those with a mobile telephone only, or those who are ex-directory are excluded unless the researcher specifically finds ways of generating telephone numbers for these groups. Anecdotally, response rates to telephone surveys in Ireland and the UK have diminished over recent years due to saturation of householders with market research company surveys. Non- response has been increasing in other countries also. In the United States, one of the largest, ongoing RDD telephone surveys, the Behavioral Risk Factor Survillance System (BRFSS) has noted a decline in response rates, from 63% in 1996 to 51% in 2001. The use of advance letters in one of the methods BRFSS has adopted as a mean of improving response rates (Link & Mokdad).

This paper compares the methods and outcomes of three large telephone surveys, conducted on the island of Ireland from 2000 to 2005. We describe the advantages and disadvantages of the different approaches used and discuss the particular difficulties encountered.

## Methods

Three telephone surveys were conducted in collaboration with the UCD School of Public Health and Population Science, University College Dublin, Ireland from 2000 to 2005. All were quantitative population-based surveys of random samples of the general public and examined health-related attitudes and behaviours. *Survey 1 *[6] examined the epidemiology of acute gastroenteritis in Northern Ireland and the Republic of Ireland. *Survey 2 *[4] assessed attitudes and behaviours of a regional population in the Republic of Ireland to heart health. *Survey 3 *[5] examined knowledge and attitudes to blood donation in Northern Ireland and the Republic of Ireland

We examined the methods used in each survey for telephone number sourcing and generation as well as respondent selection. To permit direct comparison between the three surveys, all outcome measures, including total telephone numbers dialled, contact rates, respondent eligibility status, completion rates, refusals and co-operation rates, were calculated in accordance with guidelines from the American Association for Public Opinion Research (2000) [10]. Factors potentially influencing survey response rates, such as questionnaire length and subject matter, were considered. We calculated the total cost of each survey inclusive of set-up, database expenditure, interviewer salaries, phone costs, data entry costs (if applicable) and subsequent unit cost per completed interview. Noteworthy issues as well as problems encountered during set-up, piloting, data collection and analysis were documented.

## Results

### Comparative methodology

Table 1 gives a detailed overview of the methods used in each of the three surveys. Conducted from December 2000 to January 2005, the study periods varied in length from 6 weeks to 12 months.

In surveys 1 and 3, the hundred-bank method [11] was used to generate telephone numbers. This method generates one hundred new telephone numbers from each stem (prefix) identified from the National Telephone Directory by assigning the numbers from 00 to 99 to the end of each stem (e.g. 01-1234200 to 01-1234299). While this method allows the inclusion of both listed and ex-directory residential telephone numbers in the sampling frame, ineligible fax numbers, business lines, invalid and unassigned telephone numbers are also generated. In contrast, survey 2 used the 'National Telephone Directory Database' [12]. This database was used to search for specific regional prefixes and address descriptors in order to identify all residential landline telephone numbers within the health board region of interest. As this database did not include ex-directory numbers, the sampling frame was less representative. However, as non-household telephone numbers (such as fax numbers or business lines) were also absent, the proportion of ineligible telephone numbers dialled was reduced, resulting in a higher contact rate. No survey included mobile telephone numbers in the sampling frame.

Surveys 1 and 3 used random digit dialling (RDD) [13,14] to contact households. Within each household, respondents were then randomly selected by asking to speak to the person who was next to celebrate a birthday in the household [15]. This simple technique is a commonly used to overcome the respondent selection bias associated with administering the survey to the household member most likely to answer the phone. Survey 2 surveyed the first adult contact and was therefore biased in favour of the person who answered the telephone.

Surveys 1 and 3 made up to four contact attempts to each randomly sampled telephone number. Survey 2 made only one contact attempt per telephone number. Survey 2 was conducted all day Monday to Saturday and lasted typically about 15–20 minutes; surveys 1 and 3 were approximately 10–12 minutes in length and were conducted at off-peak hours. Survey 1 was conducted by the traditional telephone to paper method, with associated data entry costs, whereas surveys 2 and 3 were done by computer- assisted telephone interview (CATI).

In survey 2 a quota technique (by quota technique we mean we pre-defined the total sample size to be broken down by age and sex groups to match the distribution of the target population) was used to ensure the sample resembled the census population structure for the region. The age-sex distribution that would occur in a representative population sample (e.g. males 45–60 formed X% of the population) was worked out *a priori *and once enough respondents of each distribution (the quota) was reached, no further respondents of that age-sex stratum were interviewed. Each age-sex stratum quota was filled. Because the sample size was designed to be representative weighting was not used in the analysis.

### Survey outcomes

Table 2 details outcome measures for the three surveys. The proportion of total telephone numbers dialled that identified an eligible respondent was 52.9% in Survey 1, 50.2% in Survey 2 and 31.8% in Survey 3. The major reason for this disparity was an increasing number of residential household non-contacts (e.g. residential answering machines) in each subsequent survey, rising steeply to 21.0% of dialled numbers in survey 3. Conversely, the proportion of dialled numbers of unknown eligibility (e.g. calls which rang out, were engaged or where phone problems occurred) rose from 23.0% (Survey1) to 30.0% (Survey 2) to 62.7% (Survey 3). The refusal rate was highest in survey 2. This regional survey, conducted on behalf of the health board, had the longest completion time (Table 1).

Males, particularly those in the 25–44 age group were underrepresented, in surveys 2 and 3. This may be the population who have transferred to mobile phone only usage. Targeted calls where the interviewer requested to speak to a male person in the household, and use of follow-on calls to their mobile phones were required to augment responses from this group.

Costs were calculated for each survey (Table 3). The unit cost of a completed survey was €4.48 in Survey 1, €6.65 in Survey 2 and €15.65 in Survey 3. The major reason for differences in survey costs were relatively high interviewer salaries in Survey 3; costs not off-set by the lower per unit phone charges availed of by the institution. The high non-contact rate and unknown eligibility in Survey 3 meant more interviewer time per completed interview.

## Discussion

This review highlights telephone surveys as a useful tool for collecting information on health related attitudes and behaviours. As in other European countries, the majority of private households in the Republic of Ireland (85%) and Northern Ireland (94%) have a land- line telephone [16], making most persons accessible using this methodology. As telephone surveys can be conducted centrally, the population coverage can be more extensive than face-to-face surveys and the cost per interview lower than for postal or face-to-face surveys. This review signals a possible ongoing decline in the utility of land-line only surveys with the increase use of ex-directory residential numbers, the increasing prevalence of mobile telephone only users, increasing consumer resistance to telephone surveying and difficulty in reaching consumers, possibly due to internet usage, these issues point towards increased potential for under-representativeness in landline surveys in the future.

### Representativeness

In the past decade, the increased use of mobile phones in the Republic of Ireland has been associated with a reduction in land-line coverage and 15% of households within the Republic of Ireland do not possess a land line. Currently 82% of adults in the Republic of Ireland have mobile phones [17] and with 24% having mobile phones only, a high rate being observed in certain groups such as students and younger adults living alone. One difficulty of including mobile telephone number in surveys is that no electronic or hard-copy listing exists for mobile telephones numbers, which rules out accessing existing numbers or generatating new ones from existing stems. If mobile telephone numbers are contacted, high roaming costs may be incurred by respondents travelling cross-border or outside the country at the time of the survey; and once contact has been made, the selection of one contact per household is impossible. Households without land- line telephones or mobile phones are proportionately more prevalent among socio-economically disadvantaged groups and older people, raising issues of representativeness among these sub-groups. The hundred-bank method overcomes the problem of ex-directory numbers not being included, thereby improving representativeness. However, because it generates fax, business and non-existent invalid numbers, interviewers in surveys 1 and 3 had to dial more numbers overall that those in survey 2 to obtain the required number of completed surveys, affecting time and costs. Some researchers have suggested the auxiliary method of using residential directory-listed status of the telephone number to check prior to dialling as a means of optimising interviewer time [18]. Other automated procedures to remove a portion of the business and non-working numbers before they are released to interviewers for calling have been widely used in the United States [19]. More recently, a method of over-sampling of household numbers has been used, using a disproportionate stratified sample [20].

Many phone survey organisations do post-survey weighting which implies that a proportionally representative sample is rarely achieved [21]. The problem occurs either because the sampling procedures (telephone number selection and/or respondent selection) or differential non-cooperation distorts the sample distributions. All of the surveys described in this paper reported a lower proportion of male compared to female respondents, which impacts on representativeness.

### Comparison of outcomes and costs

Survey 3 reported a high percentage of engaged tones (unknown eligibility) relative to the other surveys. Possible contributory factors include the increased use of caller ID and home internet use which has increased by 16% in the Republic of Ireland in 2004 from 2003 [17].

In terms of outcomes rates there was a noteworthy difference observed between response rates which varied between 58.6% (survey 1), 21% (survey 2) and 17.7% (survey 3). Indeed contact and co-operation rates appear to have declined over the time period of the three surveys. Considering that surveys 1 and 3 were very similar in terms of number generation and randomisation, factors external to methodology may account for this reduction in response rates. These factors may include saturation of good will of consumers due to greater market research using telephone surveys. Recent legislation [22] gives land-line owners the option of opting-out of market research, which will further reduce the pool of the population available to be contacted. While these new regulations do not apply to academic researchers, the move indicates an increasing unwillingness of householders to be contacted for surveys.

In terms of refusal rates surveys 1 and 3 performed best with refusals of 10.7% and 8% respectively, compared with study 2 at 18.6%. A possible explanation for this may be that the study took considerably longer to complete than the other two surveys. In survey 3 refusals were high in Northern Ireland in the first week of the survey, which took place in mid-July. This is a time of political tension in the region, with sectarian conflict between the Catholic and Protestant communities occuring during the annual Orange marching season. Response rates improved thereafter.

Unit cost per survey was highest in survey 3 with highest costs for set-up and database creation, and relatively very expensive interviewer costs. Phone charges were lower in survey 3 as the calls were made from an institution, which has a competitive rate from its service provider.

### Respondent issues and complaints

Complaints or dissatisfaction expressed by respondents were documented. Despite carefully worded introductions read out by interviewers at the outset explaining the nature of the survey, the name of the organisation carrying it out, and the random method of selecting phone numbers, the most frequently asked question by respondents was "where did you get my number?" Some respondents expressed annoyance with the Blood Transfusion Services or the Health Board at the start as they presumed numbers had been provided from these bodies. Most were content to partake when it was explained to them again that their number had been generated randomly or taken from the telephone directory. A number of specific questions were identified as being more likely to lead to break-offs (where the respondent hung up the telephone). In this survey the questionnaire had been devised based on two previously validated questionnaires addressing similar issues in the US [23,24]. Despite a preliminary pilot and an actual pilot being conducted on the questionnaire before the survey where no issues regarding a particular question arose during the actual survey one question "how many people live in the house at present?" which was part of the respondent selection process in Survey 3, seemed to arouse suspicion in some respondents about the threat of burglary. It was decided to omit this from the survey at the piloting stage in Survey 3. A number of respondents were reluctant to answer in Northern Ireland about schooling level, possibly because lack of third level education was perceived as occurring in lower socio-economic classes. Overall three complainants in Survey 2 phoned the host institution to check that the survey was valid or to complain about being contacted or the lateness of contact.

### Interviewer issues

Survey 1 was conducted by eight in-house interviewers. In study 2 the interviews were conducted by contract interviewers who were supplied by a commercial company. In study 3 experienced market interviewers where possible, and a number of inexperienced interviewers who subsequently received training, were employed for the duration of the data collection (total 8). An issue reported on previously [25,26] was noted by the interviewers in survey 3- they reported higher refusals for interviewers with Republic of Ireland accents phoning Northern Ireland, and vice versa. The interviewers stated that some respondents seemed suspicious of their accents and suggested that they would have had less refusals if they had been phoning with an indigenous accent.

## Conclusion

Our report illustrates that telephone surveys may not be as effective as they were a number of years ago. Major emerging drawbacks highlighted by this review are the reduction in contact rate of the general population by land-line telephone use alone, and the phenomenon of consumer fatigue where those contacted refuse to participate in phone interviews. Changes in telephone usage patterns are occurring at a rapid pace in the Republic of Ireland and Northern Ireland. The increasing trend towards mobile-phone-only households, particularly in the Republic of Ireland sets a new challenge for researchers. While the rising cost of obtaining completed interviews has been demonstrated by this review, nonetheless costs per completed telephone interview remain low relative to interviewer administrated surveys.

Strategies must now be considered to maximise the utility of phone interviews. Researchers wishing to address the issue of declining response rates such consider using multimode techniques. These are generally conducted by using different methods of data collection simultaneously (e.g. Computer-Assisted Telephone Survey (CATI), Computer-Assisted Self Interviews (CASI), paper and online web surveys as well as mobile phones). One survey [27] shows that the response rate from multimode techniques (web 41%; paper 31%; overall response rate 72%) is greater than using one single method of data collection. In conducting a multimode method of data collection, participants can and should be offered the method of participation which suits them best i.e. web, postal or telephone. This should increase the overall response rate and at the same time establish the genuineness of responses given.

Caveats are the lack of available mobile phone listings, and the possibility of continued consumer fatigue leading to non-response, as mobile phone users may characteristically lead busier lives. The recent legislation [22] reducing permitted commercial marketing by phone may reduce consumer fatigue, and a possible increase in response to research surveys may be a welcome outcome. Incentives may increase response, particularly if consumers felt that public health or research would be aided by their participation. The use of the quota system, with age-sex distribution matching, may mitigate against poor response from one sector (e.g. young males) of the population.

The challenge for researchers in a society where household compositions are changing fairly rapidly and where the number of adults in households is increasing, is to ensure the sampling distribution is as representative as possible. It is now time to adapt the telephone survey approach to maximise representativeness and response, and further surveillance and ongoing assessment of the value of telephone surveys is required into the future.

In conclusion researchers undertaking telephone surveys need a detailed understanding of the telephone network in that given region and who they are interested in studying. This will allow the researcher to ascertain who is being included and equally important who is being excluded and then decide whether this exclusion is important to the overall study or not. Hence one methodology for telephone surveying may not be globally applied and researchers need to pay attention to theses details.

## Competing interests

There are no conflicts of interest for any of the authors in relation to this work. The lead authors had full access to all the data in the study and had final responsibility for the decision to submit for publication.

## Contributions of authors

Dr. Mairin Boland had the original idea for the review and had substantial contributions to the design of the paper and to the acquisition, analysis and interpretation of the data. In addition Dr. Boland was involved in drafting the manuscript and revising it for intellectual content.

Dr. Mary Rose Sweeney had substantial contributions to the design of the paper and to the acquisition, analysis and interpretation of the data. In addition Dr. Sweeney was involved in drafting the manuscript and revising it for intellectual content.

Dr. Anthony Staines had substantial contributions to the design of the paper and to the acquisition, analysis and interpretation of the data. In addition Dr. Staines was involved in revising it for intellectual content.

Dr. Elaine Scallon was involvement in the design concept and was the lead investigator of one of the surveys reviewed. She also reviewed the final manuscript and contributed intellectual content.

Mary Harrington was the lead investigator of one of the surveys reviewed. She also reviewed the final document for intellectual content.

## Pre-publication history

The pre-publication history for this paper can be accessed here:



## Figures and Tables

**Table 1:** Methodologies employed in 3 telephone surveys between 2000–2005.

	**Survey 1**	**Survey 2**	**Survey 3 **
Topic under survey	Frequency of gastroenteritis on the island of Ireland	Cardiovascular health promotion: attitudes and behaviours in one Irish health board region	Attitudes, perceptions and knowledge of the population of on the island of Ireland to blood transfusion
Data collection period	Dec 2000 – Nov 2001	Sept – Oct 2003	July 2004 – Jan 2005
Data collection duration	12 months	6 weeks	7 months
Region	ROI^†, ^NI^‡^	Health Board Area in ROI	ROI, NI
Telephone number sourcing	Hundred-bank method	Telecom Eireann National Directory Database 2003	Hundred-bank method
Household randomisation	Next birth-date	First adult contact	Next birth-date
Attempts to make contact	Up to 4 calls	1 call	Up to 4 calls
Method of survey	Telephone to paper	CATI*	CATI*
Length of questionnaire	10 minutes	15–20 minutes	10–12 minutes
Age of respondents	Over 12 years	Over 18 years	Over 16 years
Schedule of interviews	Weekday evenings and all day Saturday	All day during week and Saturday	Weekday evenings and all day Saturday
Completed sample size	9,600	1,500	4,216
Total numbers generated/extracted	22,281	75,893	55,982
Contact attempts	45,603	6,893	32,786

† ROI = Republic Of Ireland

‡ NI = Northern Ireland

*CATI = computer assisted telephone interview

**Table 2:** Outcome measures and comparison of percentage response rates from surveys 1, 2 and 3 calculated as per AAPOR definitions [9]

		**Survey 1**		**Survey 2**		**Survey 3**	
		n	% of numbers dialled	n	% of numbers dialled	n	% of numbers dialled
A	Eligible*	**11,782**	**52.9%**	**3,425**	**50.2%**	**10,401**	**31.8%**
	Completed interviews	9,903	44.5%	1,538	22.3%	4,216	12.9%
	Refusals	1,815	8.1%	1,015	14.9%	2,472	7.5%
	Non-contact	64	0.29%	826	12.1%	3,709	21.0%
B	Ineligible**	**5,378**	**24.1%**	**1,351**	**19.8%**	**1,821**	**5.5%**
C	**Eligibility unknown †**	**5,121**	**23.0%**	**2,042**	**30.0%**	**5,422**	**62.7%**
	Total telephone numbers dialled‡	22,281	(100%)	6,818	(100%)	17,644	(100%)
							
	Responserate (percentage)	58.6%		28.1%		17.7%	
	Contactrate (percentage)	69.7%		47.5%		32%	
	Co-operation rate (percentage)	84.1%		45.5%		40.5%	

* Eligible calls included cases where an interview was completed, refusal occurred, non-contact at a residential household (e.g. residential answering machine) or where respondents were unable to complete the interview due to language difficulties or disability.

** Ineligible calls included non-residential numbers (such as business or fax lines) and telephone numbers which were invalid (both confirmed and suspected).

† Calls where eligibility was unknown were recorded separately. These were mostly calls which rang, but were not answered, rang out or were engaged, or where phone problems occurred.

‡ This differs from contact attempts (Table 1) as it excludes those numbers that were redialled. In survey 1 all telephone numbers generated were used. In surveys 2 and 3, sampling of extracted or generated numbers respectively was undertaken so only a proportion were used for dialling.

**Response Rate **(minimum response rate) is the number of complete interviews divided by the number of interviews (complete plus partial) plus the number of non-interviews (refusals and break-off plus non-contacts plus others) plus all cases of unknown eligibility (unknown if housing unit, plus unknown, other).

**Contact Rate **measures the proportion of all cases in which some responsible member of the housing unit was reached by the survey.

**Co-operation Rate **is the proportion of all cases interviewed of all eligible units ever contacted.

Co-operation rate = C/C [C + R +O]* 100

Where C = completed interview

R = Refusal

O = other (not completed because of language barrier or sickness/disability.

9,903/[9,903 + 1,815 + 64]*100

= 84.1%

**Table 3:** The total and per unit cost of surveys 1, 2 and 3 conducted between 2000–2005.

	**Survey 1**	**Survey 2**	**Survey 3**
Set-up and database	€ 3,810	€1,419	€ 8,870
Interviewer salaries	€27,940	€6,918	€55,707
Phone charges	€5,067	€1,794	€1,500
Data entry	€ 7,620	0	0
Total cost	€44, 437	€10,131	€66,077
**Unit cost per completed interview**	**€4.48**	**€6.65**	**€15.65**
